# CDCDB: A large and continuously updated drug combination database

**DOI:** 10.1038/s41597-022-01360-z

**Published:** 2022-06-02

**Authors:** Guy Shtar, Louise Azulay, Omer Nizri, Lior Rokach, Bracha Shapira

**Affiliations:** 1 Ben-Gurion University of the Negev, Department of Software and Information Systems Engineering, Beer-Sheva, 8410501 Israel; 2 Universidade Federal Fluminense, Instituto de Biologia, Niterói, 24220900 Brazil

**Keywords:** Computational biology and bioinformatics, Drug discovery

## Abstract

In recent years, due to the complementary action of drug combinations over mono-therapy, the multiple-drugs for multiple-targets paradigm has received increased attention to treat bacterial infections and complex diseases. Although new drug combinations screening has benefited from experimental tests like automated high throughput screening, it is limited due to the large number of possible drug combinations. The task of drug combination screening can be streamlined through computational methods and models. Such models require up-to-date databases; however, existing databases are static and consist of the data collected at the time of their creation. This paper introduces the Continuous Drug Combination Database (CDCDB), a continuously updated drug combination database. The CDCDB includes over 40,795 drug combinations, of which 17,107 are unique combinations consisting of more than 4,129 individual drugs, curated from ClinicalTrials.gov, the FDA Orange Book^®^, and patents. To create CDCDB, we use various methods, including natural language processing techniques, to improve the process of drug combination discovery, ensuring that our database can be used for drug synergy prediction. Website: https://icc.ise.bgu.ac.il/medical_ai/CDCDB/.

## Abstract (summary)

**Table:** 

Measurement(s)	drug combination effect modeling • drug combination effect modeling
Technology Type(s)	Text mining • Clinical Trials Informatics System
Factor Type(s)	Medicine
Sample Characteristic - Organism	Homo sapiens

## Background & Summary

**Fig. 1:**
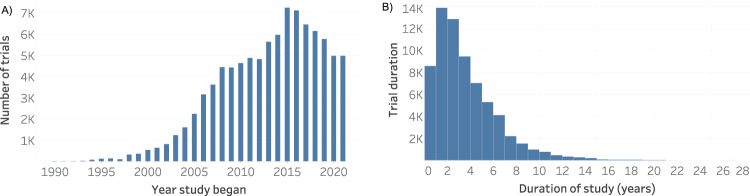
Drug combinations in clinical trials in CDCDB, version of 19th of April 2022: (**A**) Distribution of drug combination clinical trials over the years. (**B**) Duration in years of drug combination studies over the years.

Drug combinations present many advantages, mainly for treating multi-factorial diseases, where more than one genetic pathway is involved, such as cancer^1^, obesity^2^, and hypertension^3^, as well as autoimmune disorders^4^, and cardiovascular diseases^5^. Together with the methodological benefits of computational methods, it became possible to change the one-drug-one-target paradigm to the multiple-drugs-multiple-targets paradigm. In this paradigm, two or more drugs are used to treat diseases caused by altering more than one pathway. For example, infections caused by resistant bacterial strains are usually treated with therapies that combine multiple drugs for different targets because they have greater efficacy and they can decrease microorganisms’ immune systems^6^. Thus, due to the increased efficacy in treating complex diseases over the one-drug-one-target paradigm^7^, the number of drug combination studies has grown over the last 30 years (Fig. 1).

Despite the advantages of drug combinations, it is challenging to evaluate alterations in drug pathways and avoid overlapping toxicity^8^. *Drug-drug interaction* (DDI) is a common issue in polypharmacy and combined therapies^9^. In this sense, pharmacodynamics (PD) and pharmacokinetics (PK) allow a better understanding of DDI. The former refers to how the active substances move through the body, while the latter refers to what the drugs provoke when the target is achieved, i.e., the biological activity. The ADME (absorption, distribution, metabolism, and excretion) parameters and “disease module” evaluations provide insights about PK and PD and how drugs interact using disease modules^7^. Thus, when developing a new drug combination, it is fundamental to find the optimal balance between the highest synergistic effects while keeping the side effects to a minimum^10^.

Traditionally, drug combination discovery relies on experts who assess and ideate new effective drug combinations. This is a time-consuming, challenging, and complex task, as the search space for drug combinations consists of thousands of candidate drugs, thousands of potential diseases, and the need to determine the dosage for each drug. In addition, this method of drug combination discovery is not systematic and largely depends on the opinions of experts. *High-throughput screening* (HTS) enables the simultaneous measurement of the sensitivity of hundreds of cell lines to many drug combinations^11^. However, there are limitations in terms of the amount and speed of screening due to cost considerations and the need to perform physical experiments. The datasets produced using HTS are only based on *in-vitro* experiments, and most databases are restricted to cancer therapy^12–15^. Therefore, there is a need for both *in-silico* models to predict the efficacy of drug combinations and a virtual screening process that will accelerate the process and reduce the manual labour required. However, such models and processes rely on an extensive, up-to-date database based on experiments.

Considering all the difficulties when developing new drug combinations, it is partially motivated by intellectual property regulations, in which the patentee has the right, for a limited period, to prohibit third parties from producing, selling, or exporting the claimed product or process without the patentee’s consent^16^. Patents are used to protect products and processes with high innovative potential and are fundamental in high-risk fields, such as the pharmaceutical industry. Although patents could be a valuable data source for drug combinations, they have barely been used in previous drug combination databases.

Existing databases for drug combinations include the DREAM (AstraZeneca) database, which is based on experiments and consists of 11,576 experiments from 910 drug combinations for 85 cancer cell lines^17^. The DrugCombDB database contains 448,555 combinations of 2,887 individual drugs from HTS experiments and is curated from many other sources such as NCI-ALMANAC, and the literature^18^. For antifungal therapy, there is a database with 5,518 drugs forming 8,128 combinations tested against 242 strains, resulting in 492,126 samples^19^. A number of tools were introduced to aid in analyzing synergistic drugs from dose-response data of two or more drugs. SynergyFinder^20^ is an interactive tool for the analysis and visualization of drug combination screening data. CImbinator^21^ (web service is offline at the time of writing this manuscript) attempts to quantify the effects of drug combinations utilizing both the frequently used median effect equation and sophisticated mathematical models. After evaluating and ranking potential drug combinations using an in-silico model, these tools can be used to systematically identify the optimal dosage of the drugs using dose-response data.

The *Drug Combination Database* (DCDB)^22^ was among the first databases dedicated to multi-component drugs. It contained 499 approved or investigational drug combinations, including 40 unsuccessful ones and involving 485 individual drugs, curated from over 6,000 references. The latest DCDB (Version 2.0) includes 1,363 drug combinations based on 904 individual drugs interacting with 814 target genes, curated from about 140,000 clinical studies, the FDA Orange Book®, and PubMed^10^. To the best of our knowledge, DCDB 2.0 is the largest database devoted to *in-vivo* drug combinations, and many studies on drug prediction have used this database to construct prediction models^23–25^. However, the lack of an automated process for generating the data, and the manual labour required to curate the drug combinations, is not scalable and, therefore, cannot keep up with the increasing amount of related research.

An up-to-date drug combination database is currently lacking, as the DCDB 2.0 was released in 2014, 7 years ago. Since that time many new drug combinations have been investigated in clinical trials and more are expected to be investigated in the future. To address this gap, we introduce the *Continuous Drug Combination Database* (CDCDB), which currently includes 17,107 individual combinations formed from 4,129 individual drugs and is curated automatically from ClinicalTrials.gov, the FDA orange book®, and Integrity (Clarivate Analytics)^TM^. CDCDB will be continuously updated and available for download, including the three data sources; additional data sources submitted to the authors will be considered additions to CDCDB.

CDCDB is aimed at training and validating predictive models for identifying synergistic drugs. Recently, several works focused on developing such models^26–29^. Machine learning methods can be trained to solve a binary problem, i.e. “will drug a and b be synergistic?” or to estimate multi-drug synergy metrics^30–33^. In many cases, the prediction of synergistic drug combinations can benefit from different modalities (views) of the drugs, such as the known drug-target interactions, chemical structure, chemical taxonomy, etc. This information can be obtained from relevant databases such as DrugBank or PubChem by looking up the drug identifiers provided by CDCDB. Furthermore, transfer learning can be applied to tackle the synergistic drug prediction problem^34^ by using information about one disease to learn about another disease, the relevant disease for each synergistic drug set is provided in a designated field. CDCDB enables a strong retrospective evaluation by providing a weekly snapshot of the database; a retrospective is considered more true-to-life than a holdout or cross-validation evaluation schemes^35^. In most cases, drug synergy is predicted for a pair of drugs; however, CDCDB contains information about more than two drugs prescribed simultaneously.

While CDCDB does not contain the clinical outcome explicitly, a set of synergistic drugs that appears in CDCDB is likely to have at least equivalent evidence level as an in-vitro experiment: the FDA experiments require some level of evidence to start a clinical trial. Additionally, CDCDB provides a list of references for the trials. The orange book contains information about approved drugs which guarantees strong evidence from a clinical trial. A given set of drugs that appeared in patent information indicates a solid financial incentive which in most cases implies a shred of evidence regarding the synergistic potential of the drugs.

## Methods

The CDCDB is populated in a multi-step process of collecting drug combinations from various data sources, combining, normalizing, and enriching them. The main data sources consist of:

- Aggregate Analysis of ClinicalTrials.gov (AACT) database of Clinical Trials^36^
- FDA Orange Book^37^
- Integrity (Clarivate Analytics)^TM^

In addition, related and complementary data are derived from DrugBank^38^ and PubChem^39^ to uniquely identify the drugs. Derwent Innovations Index (Clarivate Analytics)^TM^ is used to complement the data regarding patents. The Unified Medical Language Service (UMLS)^40^ is used to identify the actual drugs from free text describing interventions.

### Aggregate Analysis of the Clinical Trials ClinicalTrials.gov (AACT) database

Created as an effort of the *Clinical Trials Transformation Initiative (CTTI)*, the AACT is a tabular version of clinicaltrials.gov that is automatically extracted every 24 hours^41^. As of the time of this writing, the database contained more than 359,682 studies.

The AACT provides intervention names for each design group. The intervention name is presented in free text, which creates a challenge in identifying the PubChem and DrugBank IDs of the actual drugs used in a design group. In addition to the combination *per se*, the free text might contain the administration route, dosage information, or pharmaceutical form; moreover, some typos were manually identified, such as Valgancyclovir instead of Valganciclovir. The AACT also contains studies on small molecules that are not considered drugs, such as nitrous oxide, and combinations comprising nutraceuticals, such as vitamin E. To overcome this challenge, we extract the drugs from the text using NER (named-entity recognition) with the scispaCy 0.2.5 library^42^.

The ScispaCy library contains medical data and is supported by different electronic medical vocabularies with more than three million drug names and about 83,000 ontology entities^42^. In addition, each entity has a Type Unique Identifiers (TUIs) classification^43^. We utilize this library to remove common words in the English language, symbols, numbers, and units of measurement from intervention names, to extract the actual drug name. Furthermore, the library transforms known codes and abbreviations, such as “NSC-752” into thioguanine, “5-FU” into fluorouracil, and “MMF” into monomethyl fumarate, and it also converts codes that start with “IND” into “Investigational New Drugs”, making it easier for researchers to understand the data.

To clean the data and identify studies consisting of actual drug combinations, we use ScispaCy to select combinations classified in at least one of the following TUIs:

- T109 (Organic Chemical)
- T114 (Nucleic Acid, Nucleoside, or Nucleotide)
- T116 (Amino Acid, Peptide, or Protein)
- T121 (Pharmacologic Substance)
- T123 (Biologically Active Substance)
- T125 (Hormone)
- T126 (Enzyme)
- T129 (Immunologic Factor)
- T195 (Antibiotic)
- T200 (Clinical Drug)

In addition, for the AACT data, we remove substances classified as nutraceuticals in DrugBank^44^.

### FDA Orange Book®

The FDA Orange Book® identifies drugs approved in the U.S. and provides drug names, dosage form, route of administration, brand name, applicant, type of drug (innovator or generic), FDA approval date market status, patent information, and other relevant information. At the time of this writing, the FDA Orange Book® contained 38,615 approved drug records, both individual drugs or combinations of drugs, with different dosage forms, routes of administration, and strength. Of 38,615 approved drugs, there are 4,767 combinations, or 554 combinations of 557 individual drugs when different dosage forms and other variables are omitted.

### Patents (Integrity, Clarivate Analytics)^TM^

Integrity (Clarivate Analytics)^TM^ is a database comprising more than 574,000 compounds, most of which are covered by approximately 440,000 patents from major patent offices, including those in Europe (EP), Japan (JP), United States (US), India (IN), China (CN), and the Republic of Korea (KR), as well as the World Intellectual Property Organization or WIPO (WO). This database provides references between complementary fields, such as targets and pathways, genomics, experimental pharmacology, pharmacokinetics, pharmacodynamics, clinical studies, companies and research institutions, literature and patents.

The Derwent Innovations Index (Clarivate Analytics)^TM^, licensed by FAPESP - Fundação de Amparo à Pesquisa do Estado de São Paulo: process 2017/25364–6, which is also from Clarivate Analytics, is the largest platform focused on intellectual property, with more than three million patent applications from about 50 patent offices around the world. Using the Derwent World Patents IndexTM (DWPI), patent specialists provide the Derwent Innovations Index (Clarivate Analytics)^TM^ with an improved and simplified definition of the inventions, providing a personalized title and abstract focused on technology novelties and current information about the assignee and International Patent Classification (IPC).

### Unified Medical Language Service (UMLS)

The UMLS is a collection of medical vocabularies which is updated every three months and includes more than two million words from distinct sources and their associations^40^. Using a Python library, we map keywords, such as diseases to be treated, drug names, etc., to medical terms and Concept Unique Identifiers (CUI), based on a confidence score for the mapping, which is used to find terms more or less related to the term in question.

In this paper, we utilize the UMLS in order to clean the Clinicaltrials.gov data. Using NER, we identify the UMLS of each intervention from free text in Clinicaltrials.gov. Then, the UMLS is used to identify which entities are actual drugs and separate them from the free text.

### Drug identifier retrieval

DrugBank is constantly updated. The latest version (5.1.7) includes 14,460 drugs and 4,118 approved drugs; each drug has a unique identifier that starts with the prefix “DB” followed by five digits. For instance, DB09037 is the identifier for the monoclonal antibody (mAb) pembrolizumab.

PubChem is the world’s most extensive repository of publicly available chemical data, built from more than 700 data sources and comprising identifiers for more than 100 million chemical compounds, 260 million substances, and 260 million bioactives. It provides the drug name and additional drug-related information, such as molecular formula and structure.

Drug names are inappropriate identifiers due to different synonyms. Furthermore, using Machine Learning (ML) to predict drug-related property requires informative features that represent the drug, for example, the molecular drug structure. In order to retrieve features for the drugs, a unique and accepted identifier for the drug is needed. For these reasons, in the CDCDB, we retrieve the *DrugBank Identifier* (DBID) and the *PubChem Identifier* (CID) for each drug in the database, using algorithm 1. Since the source data contains several different names for the same drug, typos, different languages, or even drugs under investigation that are still classified by codes not recognized by Drugbank or Pubchem, there are some records in the data for which a unique identifier is missing. However, CDCDB provides the intervention name that can be used to identify each drug.

**Fig. 2:**
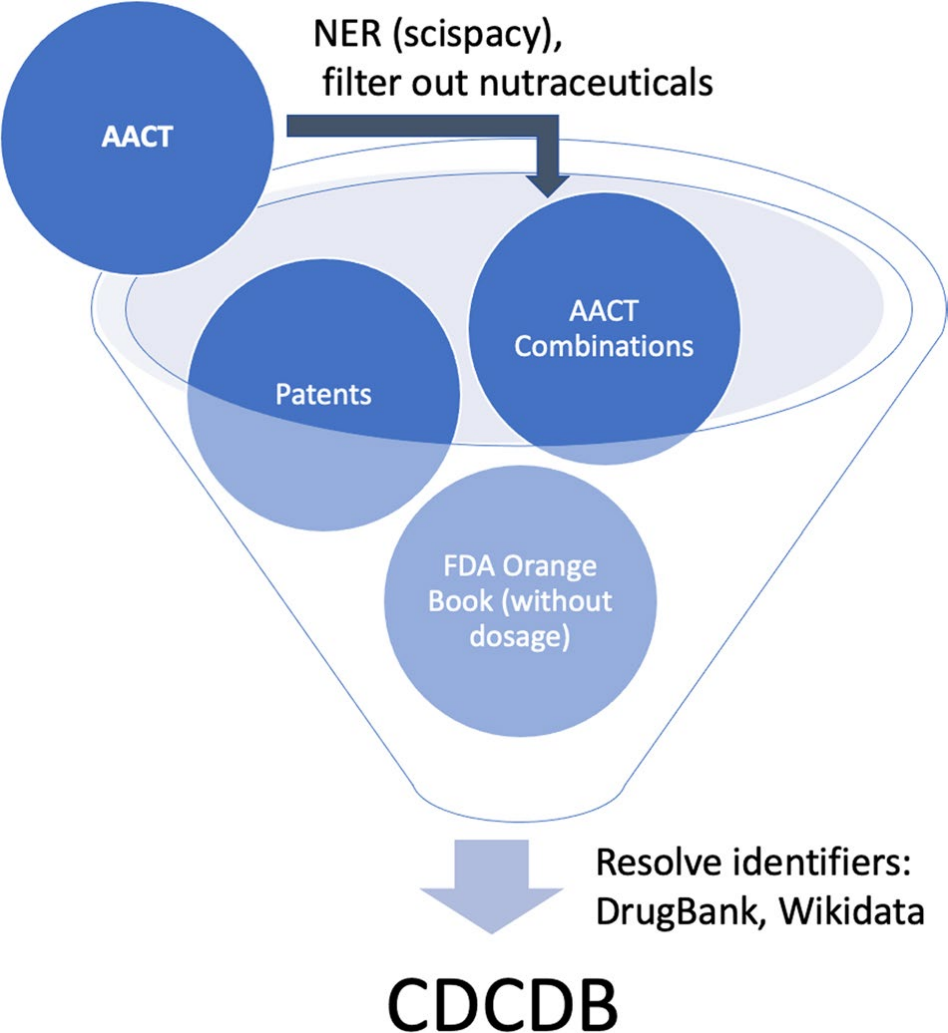
Overview of the database creation process. CDCDB is created from various sources, named entity recognition and manual filtering techniques are used to maintain the quality of CDCDB.

Due to the inherent free text in Clinicaltrials.gov, we cleaned the intervention names before running the identification algorithm; this allows us to obtain better matches between intervention name and unique drug identifier. To accelerate this process, all of the identifiers retrieval functions used a local cache mechanism. The overall process for creating CDCDB is presented in Fig. 2.

## Data Records

CDCDB is publicly accessible for download in a CSV format from figshare^45^ and from a public website (https://icc.ise.bgu.ac.il/medical_ai/CDCDB/) where it is being updated constantly by running a script once a week to create a new version. Additionally, the website keeps records of the past CDCDB versions, available for download on the “downloads” section. CDCDB consists of four groups of tables derived from the data sources mentioned above. The records are described in detail below:

1. ClinicalTrials (AACT)
2. FDA Orange Book
3. Patents (Integrity, Clarivate Analytics)^TM^
4. All combinations - unnormalized version

### Algorithm 1

Drug identifier retrieval algorithm.

The tables are described in the subsections that follow. A visual schema (ERD) of CDCDB is presented in supplementary information Fig. 1.

### ClinicalTrials.gov (AACT)

The clinical trials group of tables contains data related to studies on drug combinations identified by our database creation system. These tables include the relevant design groups (in our case, the drug combination tried on a group) and the respective metadata about the trial. This group was divided into various tables as follows. After cleaning the data, we had 17,107 combinations of 4,129 individual drugs from Clinicaltrials.gov. Each clinical study has an NCT ID (a unique identifier for the clinical trial), which is linked to the the title of the study, the names of the drugs used in the intervention, the study’s references, the conditions that the clinical trial study (and their respective MeSH terms), and one or more design group ID.

CDCDB contains the following clinical trials related tables:

- Design Groups
- Clinical Trial Studies
- Conditions
- MESH Terms
- References

The **Design Group Table** lists the different design groups used in each study. For each design group, the table contains the drugs names, identifiers, and the type of the group from the following list: “experimental”, “active comparator”, “placebo comparator”, “no intervention”, “sham comparator”, or “none given” when no information was provided. The experimental design group is the focus of the clinical trial and consist of a group of participants receiving the primary intervention. The active comparator group is the administration of an effective intervention compared with the experimental group. The placebo comparator arm is comprised of participants that receive the placebo. The no intervention arm is the group of participants who do not receive any intervention. The sham comparator group is related to a procedure or device similar to the experimental group but without active processes or components. In the case of observational studies, there is no specific division into subgroups; therefore, these groups are filled with “not applicable”.

The **Clinical Trial Studies Table** contains information for each clinical trial: study start and completion dates, overall status (not yet recruiting; recruiting; enrolling by invitation; active, not recruiting; suspended; terminated; completed; withdrawn; unknown status), phase (early phase 1; phase 1; phase 1/2; phase 2; phase 2/3; phase 3; phase 4; “not applicable” for cases of observational studies or sham comparator), enrollment (number of participants), enrollment type (actual or anticipated), number of arms (for clinical trials), number of groups (for observational studies), and, if applicable, why the study was interrupted.

The **Conditions Table** contains the conditions to be treated in each clinical trial.

The **MeSH Terms Table**, similarly to the conditions table, contains all the MeSH terms of the conditions that are treated in the study. For instance, the NCT00002594 study aims to treat brain and central nervous system tumours, and the related MeSH terms are brain neoplasms, germ cell and embryonal, nervous system neoplasms, central nervous system neoplasms, neoplasms, and Medulloblastoma. Table 1 presents the top MESH terms found in CDCDB and the number of occurrences for each of the MESH terms.

**Table 1:** MeSH terms of the clinical studies in CDCDB.

MeSH term	Occurrences
Other	17,795
Breast Neoplasms	1,153
Lung Neoplasms	903
Lymphoma	870
Leukemia	855
Carcinoma, Non-Small-Cell Lung	854
Carcinoma	728
Neoplasms	693
Multiple Myeloma	634
Neoplasms, Plasma Cell	568
Hepatitis C	440
Hepatitis	435
Prostatic Neoplasms	430
Leukemia, Myeloid	420
Diabetes Mellitus, Type 2	417
Leukemia, Myeloid, Acute	408
Diabetes Mellitus	383
Adenocarcinoma	360
Colorectal Neoplasms	351
Hepatitis A	349
Leukemia, Lymphoid	336
Melanoma	329
Myelodysplastic Syndromes	321
Syndrome	299
Leukemia, Lymphocytic, Chronic, B-Cell	290
Lymphoma, Non-Hodgkin	277
Lymphoma, B-Cell	256
Hepatitis C, Chronic	253

The **References Table** contains all of the literature directly (results references) or indirectly (references) related to the clinical trial. Of all of the references used in the trials, most references are scientific papers closely related to the drug or disease that is the focus of the clinical study; the remaining papers disclose the study conducted.

A simplified representation of CDCDB is provided in the table **Web preview** which is also available using non-programmatic access through our website. The table contains all of the combinations of drugs that appeared in any sources used to create CDCDB. The drugs are described using their name, DrugBank ID, and PubChem ID.

### The FDA Orange Book®

The **Orange Book Combinations Table** comprises information about drugs: trade and drug names, product number, application type (N for an innovative drug and A for a generic drug), TE code (therapeutic equivalence rating of generic to innovator Rx products), FDA approval date, RLD (reference listed drug with an FDA safety and effectiveness finding), RS (reference standard drugs for generic development), marketing status (RX, OTC, or DISCN), and applicant. This table also contains patent-related information: the patent number, patent submission and expiration dates, patent delist request flag (in cases in which the sponsor has requested patent to be delisted), drug substance and drug product flags (for patents in which the applicant filed the patent to claim the substance or product), and patent use code (for patents covering approved therapeutic indications).

After omitting dosage forms, route of administration, and strength, 554 individual combinations were retrieved, consisting of 561 individual drugs from the FDA Orange Book®. The DBID or CID was found for 539 individual drugs; of these, the drugs forming the most combinations are sodium chloride (64), potassium chloride (55), hydrochlorothiazide (36), magnesium chloride (34), and ethinyl estradiol (26). Only 187 combinations have patent information, and, as explained below, there are some duplicated patents and combinations. For instance, US7125873 covers both metformin hydrochloride/sitagliptin phosphate and simvastatin/sitagliptin phosphate; US9511056 covers ledipasvir/sofosbuvir, but with distinct strengths (45mg/200mg and 90 mg/400 mg). The same combination can also have a different dosage form or route, such as US7704984, for which Ethinylestradiol/Norethindrone acetate is produced in capsule or tablet form.

### Patents (Integrity, Clarivate Analytics)^TM^

The same patent can protect different drug combinations, and different patents can protect the same drug combination in different ways, such as the so-called primary and secondary patents. For instance, EP205530 covers both levonorgestrel/ethinylestradiol (Alesse®) and estradiol valerate/dienogest (Climodien®), while US20080110792 claims “a unit dosage package for a pharmaceutical formulation (...)” comprising bupropion/naltrexone (Contrave®), while US20120093889 claims “a method for affecting weight loss in a patient, comprising identifying a patient in need of weight loss, administering to the patient a layered pharmaceutical formulation comprising (...)” bupropion/naltrexone (Contrave®). Considering only unique drug combinations, we retrieved 14,209 patents from Integrity (Clarivate Analytics)^TM^. The CDCDB contains the following patent related tables:

- Transformed Patent Drug Table
- Patent Metadata Table
- Patent IPC Table

The **Transformed Patent Drug Table** contains for each patent ID the drug name, brand name, code name, Integrity code, molecular and cellular mechanisms, mechanism of action, phase (biological testing, preclinical, IND filed, clinical, phase 0, phase 1, phase 2, phase 3, preregistered, recommended approval, registered, launched, discontinued, suspended, withdrawn, undetermined, and not applicable), active development (yes or no), description of drug combinations (free text), and conditions to be treated (free text).

The **Patent Metadata Table** contains all patents from Integrity (Clarivate Analytics)^TM^ along with their metadata from Derwent Innovations Index (Clarivate Analytics)^TM^. This metadata includes information such as the publication date, assignee, title and abstract, claims, status (alive, dead, or indeterminate), cited and citing patents, INPADOC (International Patent Documentation) family and IPC (International Patent Category). Alive patents are active (granted or with ongoing prosecution); dead patents were not granted or were granted but expired. Otherwise, the patent is classified as indeterminate. Forward and backward citations can be used to understand knowledge’s evolution over the years, and the INPADOC family can be used to visualize in which countries a specific technology was filed.

The **Patent IPC Table** contains a mapping between Patent ID and IPCs, since the same patent can have more than one IPC. The IPC is used to indicate the patent’s categories.

### All Combinations

The unnormalized version of the database contains all of the drug combinations from the three data sources. Each row in this table has the form of combination (drug names), DrugBank ID(s), PubChem ID(s), source (either Clinicaltrials.gov, the FDA Orange Book®, or patents from Integrity (Clarivate Analytics^TM^).

## Technical Validation

When creating the database, we used a few techniques to decrease the errors in the various data sources. An expert in the biology field performed an initial manual review and defined data cleaning rules. The rules were essential for the AACT (Clinicaltrials.gov) database since the drug name is part of the intervention name, including other information (free text). To handle that, we removed common words that interfere with the algorithm’s retrieval of the drug identifiers, such as dosage information (mg, kg, mg/day, low dose, fixed), administration route (oral, sublingual, topical, vaginal, mucosal), and pharmaceutical form (tablet, suspension, troches, spray). Moreover, as described in the Methods section, we included only “complex substances” in the database. i.e., we keep only substances with more than two chemical elements; for example, we remove oxygen, nitrous oxide, etc. In order to improve the quality of the data, we employed the *NLP (natural language processing)* Python library (scispaCy^42^). With scispaCy’s NER method, we were able to classify words that represent drugs, which provided a more accurate way to identify the actual drugs in the interventions names, separating it from the free text like dosage, descriptions, trademarks. We also removed those substances classified as nutraceuticals in DrugBank.

**Fig. 3:**
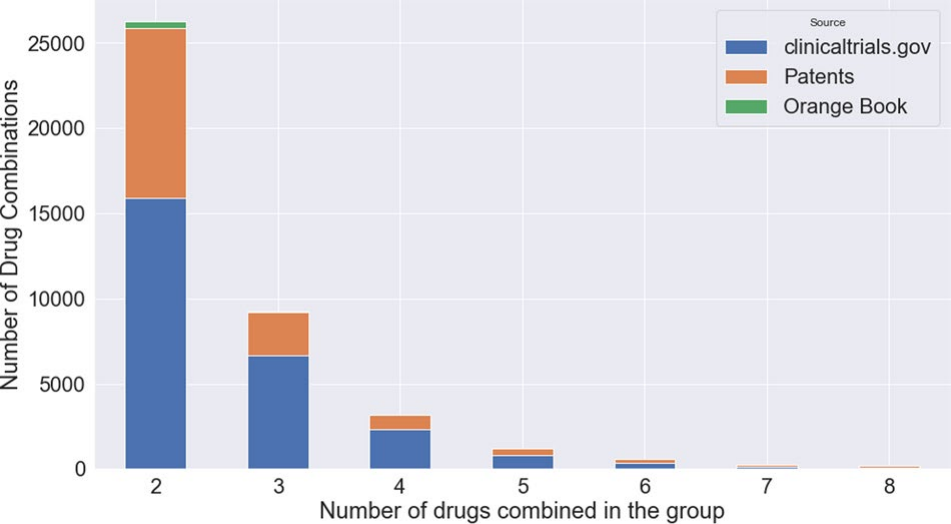
CDCDB’s sources. Amount of groups with a certain number of drugs combined in the group, distributed by data source.

Table 2 provides a comparison of existing drug combination databases. The current DCDB, as well as other five existing databases, contain drug combinations for many diseases, however, they contain fewer combinations (DCDB 2.0) or rely mainly on HTS information (the most recent DrugCombDB). Note that the large number of combinations in DrugCombDB is because drug combinations are repeated for different cell lines and dose responses; these were excluded from the CDCDB. Moreover, the impressive number of drug combinations in the TTD (Therapeutic Target Database) is because this database includes small molecules. In addition, PubMed is used as a source, so drugs that will not get market approval are included. Figure 3 presents the number of drug combinations found in CDCDB separated by the source of the combination and the number of drugs combined.

**Table 2:** Comparison of drug combination databases.

Database	Sources	Number of Combinations	Number of Individual Drugs	Therapeutic Field	Release Year
DCDB 1.0^22^	Orange Book and PubMed	499	485	Many	2010
DCDB^10^ 2.0	ClinicalTrials, Orange Book, and PubMed	1,363	904	Many	2014
ASDCD^48^	PubMed, Google Scholar, and Web of Science	210	105	Fungal	2014
NCI-ALMANAC^49^	HTS from Orange Book	5,232	104	Cancer	2017
TTD^50^	FDA, ClinicalTrials and PubMed	34,019	unknown	Many	2018
DREAM^17^	FDA, HTS experiments	910	115	Cancer	2019
DrugComb (includes mono-therapies)^51^	Public datasets, publications, user uploads	739,964 (mostly in-vitro)	8,397	Cancer, Malaria, COVID-19	2021
DrugCombDB^18^	HTS experiments, Orange Book, NCI-ALMANAC, DREAM, PubMed, and external databases	448,555 (only ∼7,000 in-vivo)	2,887	Many	2020
O’Neil *et al*.^52^	HTS experiments	583	38	Cancer	2016
NCATS Malaria Dataset^53^	HTS experiments	14,810	206	Malaria	2015
AZ-DREAM^54^	HTS experiments	910	85	Cancer	2015
Antibiotic combinations^55^	HTS experiments	210	21	Antibiotic	2006
CDCDB (current work)	AACT (clinical trials), FDA Orange Book, and Integrity (patents)	40,795 (as of Jan. 2021)	4,195 (as of Jan. 2021)	Many	**Continuous**

While there are 1,363 drug combinations in DCDB 2.0, as of January 2021, CDCDB contains 40,795 combinations, of which at least 17,107 combinations are unique, consisting of more than 4,129 individual drugs. The CDCDB was built from three distinct databases that are not solely devoted to drug combinations. In order to utilize the data from these sources, we effectively cleaned and integrated the data from ClinicalTrials.gov, the FDA Orange Book®, and Integrity (Clarivate Analytics)^TM^. Although the FDA Orange Book® includes patent information, it is restricted to drugs approved in the U.S. In contrast, the CDCDB includes patent information from Integrity (Clarivate Analytics)^TM^ and the Derwent Innovation Index (Clarivate Analytics)^TM^, which cover patents from many other patent offices.

The CDCDB’s technical validation is guaranteed mainly due to the cleaning step in which the NLP NER technique is used to identify the entities (i.e., drugs) in the free text and select them. The strength of the CDCDB derives from combining and properly cleaning data from three different data sources. The new database allows researchers to explore not only drug combinations but diverse related information and metadata. These advantages make CDCDB the most complete, accurate, and updated database that is continuously updated.

As part of our comprehensive technical validation process, we track synergistic drugs found in the latest version of CDCDB to their source. According to CDCDB, the combination of Hydrocortisone, Fludrocortisone, Letrozole, Flutamide was used in trial NCT00001521, a phase 2 trial with two arms, the trial is associated with the condition Congenital Adrenal Hyperplasia (CAH), and the mesh terms (1) Adrenal Hyperplasia, Congenital; (2) Adrenocortical Hyperfunction; (3) Adrenogenital Syndrome; and (4) Hyperplasia. To validate this information, we looked up trial NCT00001521 in clinicalTrials.gov; we found that all of the information found in CDCDB describing this trial is correct. Furthermore, the drug identifiers DB00741, DB00687, DB01006, and DB00499 are associated with the drugs used as interventions in the trial. Next, we validated a combination reported in a patent application; the combination of Flurbiprofen and Tolperisone appeared in application WO2020086046. The following DrugBank ids were found in CDCDB for these drugs: DB00712 and DB06264 correspondingly. According to the *patent metadata* table of CDCDB, the first claim of the application is “a topical pharmaceutical composition comprising tolperisone hydrochloride in combination with flurbiprofen.” From Google patents, we confirmed that the application, entitled “Topical compositions comprising tolperisone and flurbiprofen combination” makes the reported claims. Furthermore, the identifiers reported by CDCDB correspond the drug names found in DrugBank. Lastly, we manually validate a combination from the FDA’s orange book: ethinyl estradiol and norgestimate from the *orangebook combs* table with DrugBank ids DB00977 and DB00957 are recorded as part of the product “Ortho Cyclen-21” approved on Dec 29, 1989. To validate this record, we searched for the product name on Drugs.com. The information on the website confirms that the product contains a combination of female hormones, ethinyl estradiol and norgestimate. The DrugBank identifiers for both drugs were also manually validated.

To further validate CDCDB, we use it as part of a modeling task for predicting synergistic pairs of drugs for the most common condition found in clinicalTrials.gov, breast neoplasm. The model consists of an XGBoost^46^ model trained on structured drug features collected from DrugBank. The features of the two drugs are summed to represent the drug combination. We use CDCDB to conduct a retrospective analysis to evaluate the model. The model is trained on drug pairs collected until Aug 31, 2021, and evaluated on drug pairs collected until Sep 28, 2021. Equal size of negative samples is generated for the train and test sets by selecting a single positive drug that appears in the corresponding set and a random drug that did not. We report an area under the receiver operating characteristic curve score of 0.87 and an area under the precision-recall curve of 0.9 for this evaluation. A SHAP^47^ analysis was performed to gain an understanding of the model’s decisions. SHAP (SHapley Additive exPlanations) is a technique based on game theory to explain a predictive model’s output. According to this analysis, the most contributing features (supplementary information Fig. 2) are antineoplastic agents, immunomodulating agents, and cytochrome P-450 substrates. The explanation for the model’s decision for a single positive and negative combination is presented in supplementary information Figs. 3-4 correspondingly. The code for training and evaluating this model is available as a usage example of CDCDB.

## Usage Notes

To obtain the latest version of CDCDB, please visit our website at: https://icc.ise.bgu.ac.il/medical_ai/CDCDB/, where new versions of the database are automatically created weekly. Our website also includes a history (ordered by date) of the versions generated by the system over time. A usage example is also available.

## Supplementary information

Supplementary Information

## Data Availability

All of the source code for CDCDB database generation has been uploaded to GitHub: https://github.com/Omer-N/CDCDB, where it is maintained. We also provide the code for parsing and visualizing the data (see Usage Notes above).
