# Supplementary material for: CDCDB: A large and continuously updated drug combination database
Source: Sci Data. 2022 Jun 2;9:263. doi: 10.1038/s41597-022-01360-z (PMC9163158; doi:10.1038/s41597-022-01360-z)
Supplement: Supplementary file 1 — Supplementary Information [file 41597_2022_1360_MOESM1_ESM.pdf]

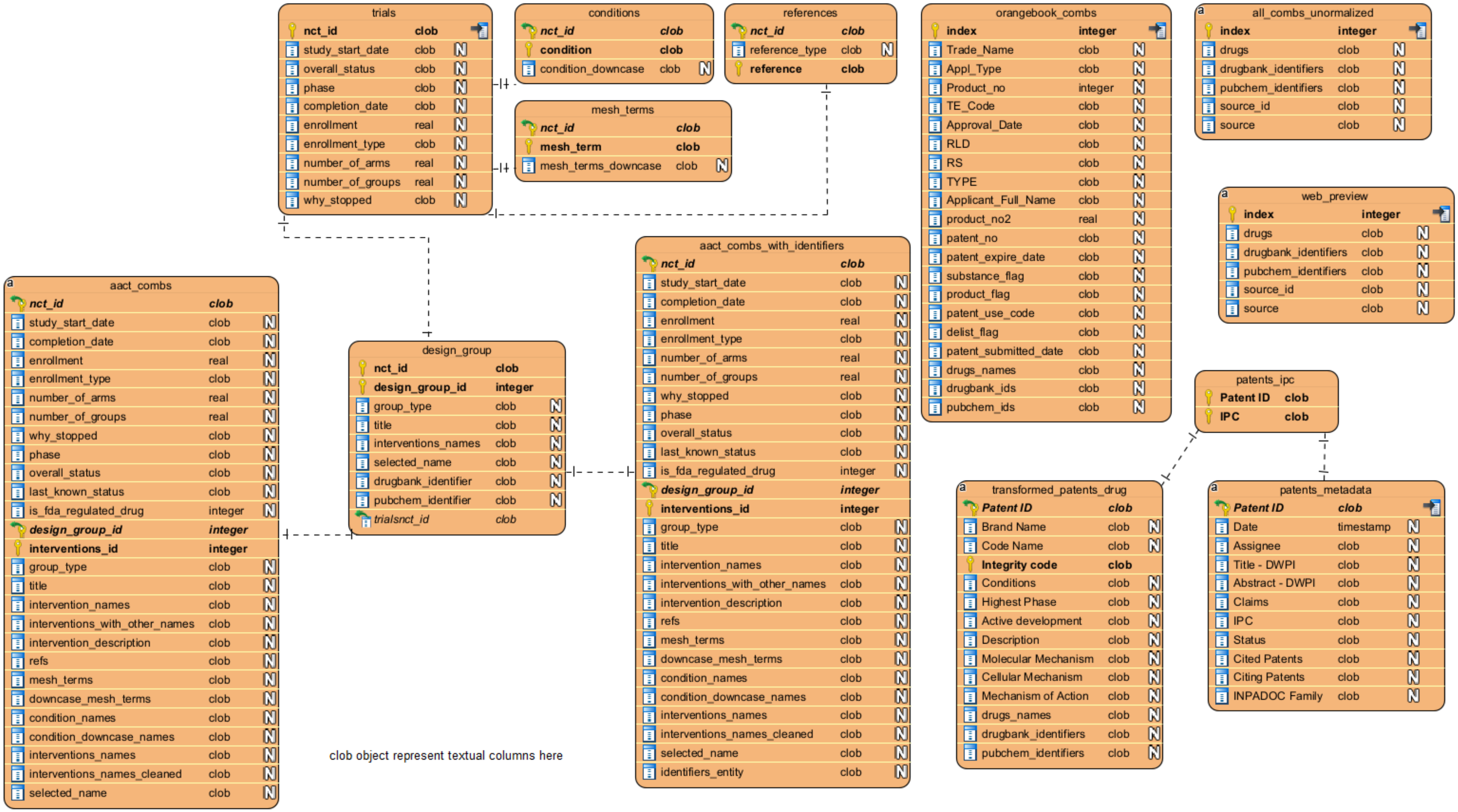

Figure 1: CDCDB schema.

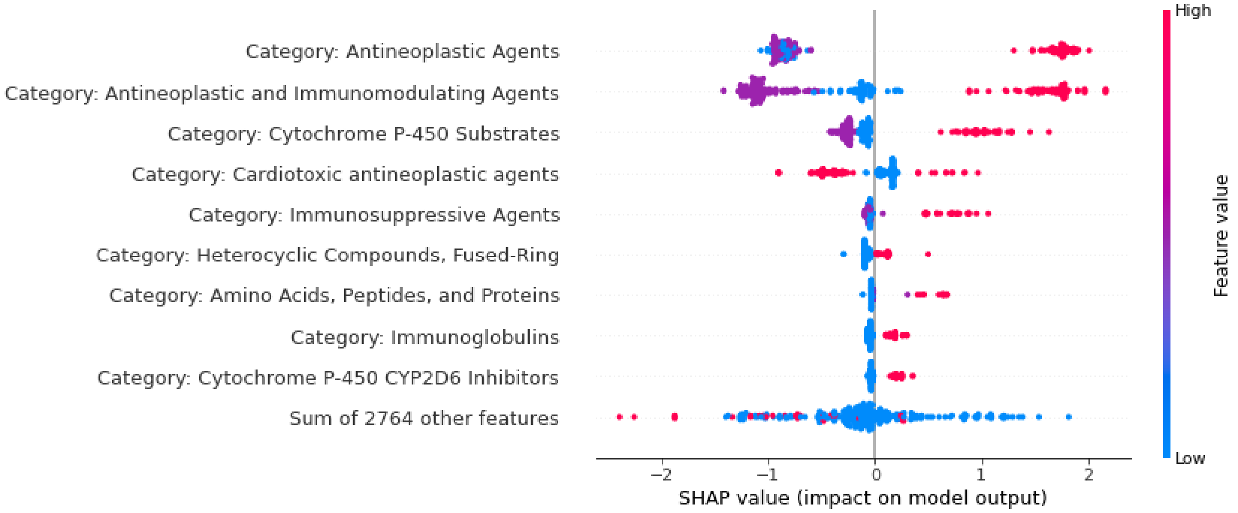

Figure 2: SHAP summary plot of an XGBoost for predicting synergistic drug pairs based on structured data collected from DrugBank. Features are ranked from top to bottom based on importance, each point represents a sample in the test set.

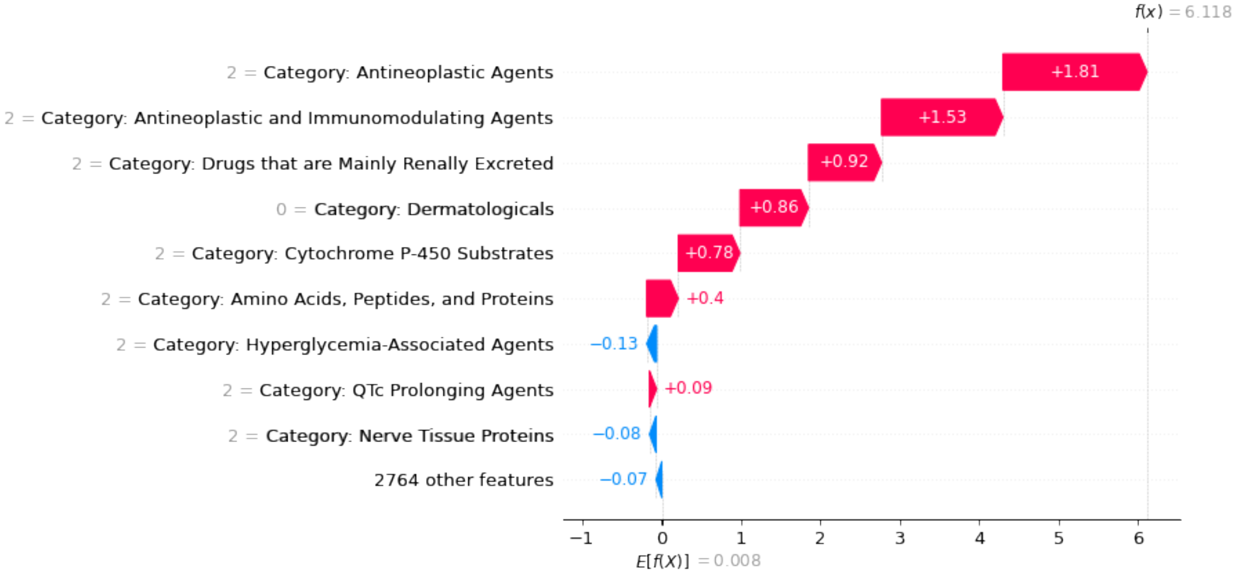

Figure 3: a decision plot of an individual positive synergistic pair predicted by an XGBoost model based on structured features collected from DrugBank. Features are ranked from top to bottom based on importance.

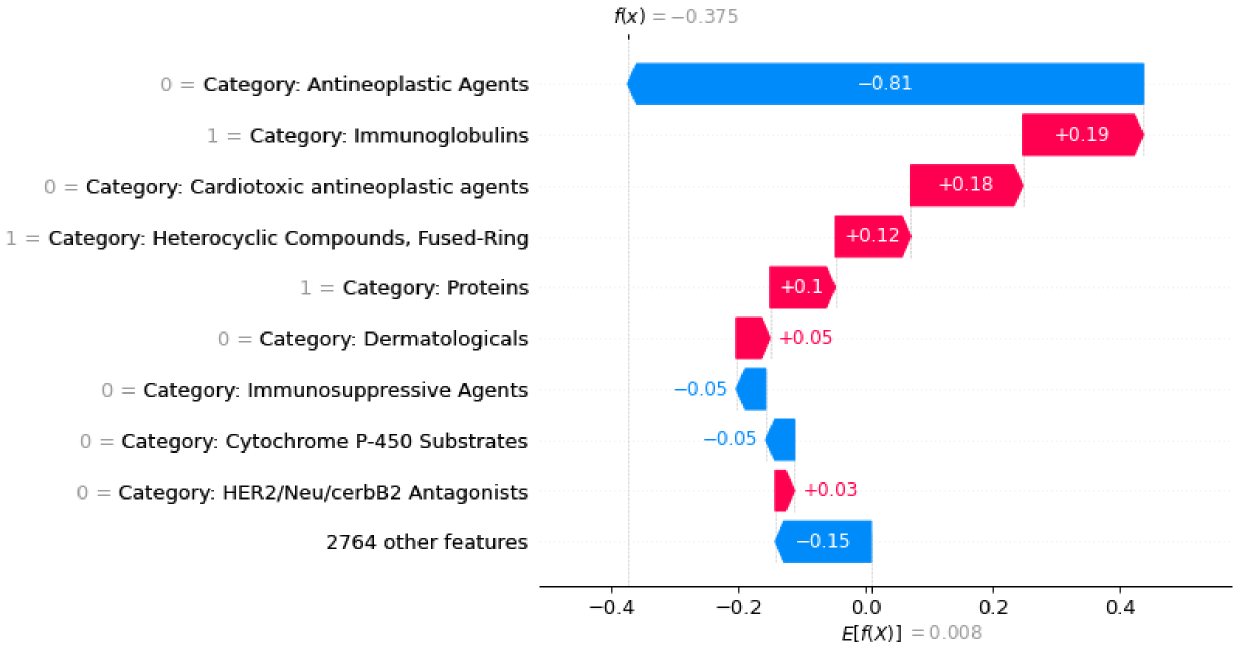

Figure 4: a decision plot of an individual negative, non-synergistic pair predicted by an XGBoost model based on structured features collected from DrugBank. Features are ranked from top to bottom based on importance.
